# Comparative hydrodynamic characterisation of two hydroxylated polymers based on α-pinene- or oleic acid-derived monomers for potential use as archaeological consolidants

**DOI:** 10.1038/s41598-022-21027-4

**Published:** 2022-11-01

**Authors:** Michelle Cutajar, Fabricio Machado, Valentina Cuzzucoli Crucitti, Susan Braovac, Robert A. Stockman, Steven M. Howdle, Stephen E. Harding

**Affiliations:** 1 School of Biosciences, National Centre for Macromolecular Hydrodynamics (NCMH), University of Nottingham, Sutton Bonington, LE12 5RD UK; 2 School of Chemistry, University of Nottingham, University Park Nottingham, NG7 2RD UK; 3 Instituto de Química, Universidade de Brasília, Campus Universitário Darcy Ribeiro, Brasília, DF 70910-900 Brazil; 4 Centre for Additive Manufacturing, Department of Chemical and Environmental Engineering, Faculty of Engineering, University of Nottingham, Nottingham, NG7 2RD UK; 5 Museum of Cultural History, University of Oslo, Kabelgata 34, 0580 Oslo, Norway

**Keywords:** Biophysics, Biotechnology

## Abstract

The Oseberg Viking ship burial is one of the most extensive collections of Viking wooden artefacts ever excavated in Norway. In the early twentieth century, many of these artefacts were treated with alum in order to preserve them, inadvertently leading to their current degraded state. It is therefore crucial to develop new bioinspired polymers which could be used to conserve these artefacts and prevent further disintegration. Two hydroxylated polymers were synthesised (TPA6 and TPA7), using α-pinene- and oleic acid-derived monomers functionalised with an acrylate moiety. Characterisation using biomolecular hydrodynamics (analytical ultracentrifugation and high precision viscometry) has shown that these polymers have properties which would potentially make them good wood consolidants. Conformation analyses with the viscosity increment (ν) universal hydrodynamic parameter and ELLIPS1 software showed that both polymers had extended conformations, facilitating in situ networking when applied to wood. SEDFIT-MSTAR analyses of sedimentation equilibrium data indicates a weight average molar mass *M*_w_ of (3.9 ± 0.8) kDa and (4.2 ± 0.2) kDa for TPA6 and TPA7 respectively. Analyses with SEDFIT (sedimentation velocity) and MultiSig however revealed that TPA7 had a much greater homogeneity and a lower proportion of aggregation. These studies suggest that both these polymers—particularly TPA7—have characteristics suitable for wood consolidation, such as an optimal molar mass, conformation and a hydroxylated nature, making them interesting leads for further research.

## Introduction

The Oseberg Viking ship burial is one of the foremost discoveries of Viking artefacts in the world. The treatment that these artefacts underwent upon excavation in the early twentieth century^1^ has unfortunately resulted in their current state of degradation. This treatment was applied to the wooden finds which had been most degraded during burial. It involved the use of hot alum (KAl(SO_4_)_2_·12H_2_O and/or NH_4_Al(SO_4_)_2_·12H_2_O) which we now know generated sulfuric acid during immersion which the wood absorbed. The acid has led to post-conservation deterioration of the remaining wood polymers resulting in the artefacts being almost destroyed^2,3^. Analyses of the alum-treated Oseberg artefacts with scanning electron microscopy (SEM) have confirmed that the secondary cell walls of the wood are more or less missing entirely^4^. Moreover, the alum did not completely penetrate the wood, resulting in a hard, alum-rich surface and a structurally weak, alum-poor core^5^. This has had a detrimental effect on the mechanical properties of the artefacts, due to crack formations between alum-rich and alum-poor zones as well as in the alum-poor core caused by shrinkage during post-treatment drying^4^. Alum-treated wooden objects are in various states of preservation and reconstruction which makes current water-based methods inappropriate to reconserve them. Due to cases like those of the Oseberg artefacts, it is imperative that we expand the currently existing toolbox of materials for treating archaeological wood.

Compounds inspired by naturally occurring materials, that is bioinspired compounds, have long generated a considerable amount of interest for such purposes^6,7^. Any wood consolidant should ideally fulfil a number of requirements such as being derived from sustainable materials, being able to form interactions with the wood and having an appropriately small molar mass^8^. Reversible treatments are considered to be the ethical ideal for conservation of culturally significant objects. However, it has been argued that in the case of the Oseberg objects, future retreatability is more important than the actual reversibility of a treatment since the objects are unlikely to survive future attempts to remove consolidants before application of a new conservation treatment^9^. Retreatment is only possible if the openings in the wood structure are not clogged with polymer, leaving space for future consolidants to pass through. As a result, any new consolidants should ideally penetrate to a sufficient depth whilst leaving room in the cells for future retreatment. As a point of reference, the molar masses of polyethylene glycol (PEG) preparations which are used in the conservation field are usually between 0.2 and 4.0 kDa^10,11^. Wakefield et al.^10^ stipulate that a molar mass of 5.0 kDa or below should be targeted for potential archaeological wood consolidants in order to maximise polymer penetration. For example, Christensen et al.^12^ have demonstrated that the uptake of chitosan by wood was shown to increase when it was depolymerised to ~ 6.0 kDa. In addition, interactions between the consolidant and wood (such as through hydrogen bonding) improve its mechanical stability. Polymer-wood interactions may also result in a lower number of hydroxyl groups in the wood cell wall, leading to increased hydrophobicity (which increases dimensional stability) and slower decay^13,14^.

The Oseberg collection is made up of highly heterogenous wood in various states of preservation and it therefore requires a diverse toolkit for its retreatment. Some of the pieces are far too fragile to be able to withstand treatment with PEG, which involves water immersion. This is because such a procedure would result in the dissolution of the remaining alum in the wood, leading to total disintegration. Other pieces have been re-constructed with various components such as metal rods, glue and plaster^4^. For these objects, there is also increased risk of damage if they are treated with an aqueous consolidant. Consequently, such artefacts should instead be preserved using consolidants in non-aqueous solvents, applied through injection, for example. Such organic-soluble consolidants which are in use in the conservation field include Paraloid™ B-72, a thermoplastic acrylic resin, and Butvar® B-98, a polyvinyl butyral-based resin. The aim for this work was to synthesise ‘greener’ alternatives to these consolidants, which can thereafter be used to treat both the highly degraded and highly reconstructed objects of the Oseberg collection.

We have recently reported on the synthesis and characterisation of an α-pinene-derived polymer which showed encouraging properties for a wood consolidant^15^. This polymer possessed hydroxyl moieties, which may enhance its hydrogen bonding potential^8^. Moreover, it had a small molar mass of 4.2 kDa and conformation analyses indicated that it had an elongated shape, both of which may increase its penetration in wood. Terpenes such as α-pinene appear to be an ideal basis for the development of such materials^16^. Not only are they abundantly available, but they can also be easily functionalised with specific chemical groups, such as hydroxyl groups, which may lead to interactions with the wood^17–20^.

As an evolution of that work, in this paper we describe the synthesis and characterisation of two other α-pinene-derived materials: a homopolymer and a copolymer with an oleic acid-based monomer. For the terpene component of the polymers, α-pinene was first converted to *trans*-sobrerol and then acrylated before polymerisation. The synthesis of sobrerol methacrylate has been previously described by Lima et al.^21^, who then used these materials to replace styrene in polyester resins. Stamm et al.^22^ have recently reported on the polymerisation of sobrerol methacrylate using both enzymatic conversion and different radical polymerisation methods. Sobrerol is a particularly interesting starting point for polymers since it is adaptable to different polymerisation techniques and post-polymerisation chemical modifications^22^.

Vegetable oils such as oleic acid are considered to be one of the most accessible and affordable resources available^23,24^, making them highly popular feedstocks for the synthesis of biobased polymers^23–28^. Additionally they generally have low toxicity^23,24,29,30^, making them very attractive to work with in conservation. In this work, oleic acid was acrylated in order to transform it into a monomer. To the best of our knowledge, a copolymer made up of these two monomers, α-pinene acrylate and oleic acid acrylate, has not yet been reported on.

Analytical ultracentrifugation (AUC) was used to characterise the size distribution and molar mass of both polymers, as it has proven to be a robust and highly accurate technique to study polymers^31,32^. Moreover, it has recently been used in the study and characterisation of existing and potential wood consolidants^10,15,33–36^. Being an absolute method it does not require a calibration standard^37^. Combining this with other techniques, such as viscometry and differential scanning calorimetry (DSC), has also allowed us to get an estimate of the shape and glass transition temperature (*T*_g_) of the polymers. This will enable us to more accurately predict whether the synthesised polymers would make successful wood consolidants.

## Results and discussion

### Monomer synthesis from α-pinene

**Figure 1:**
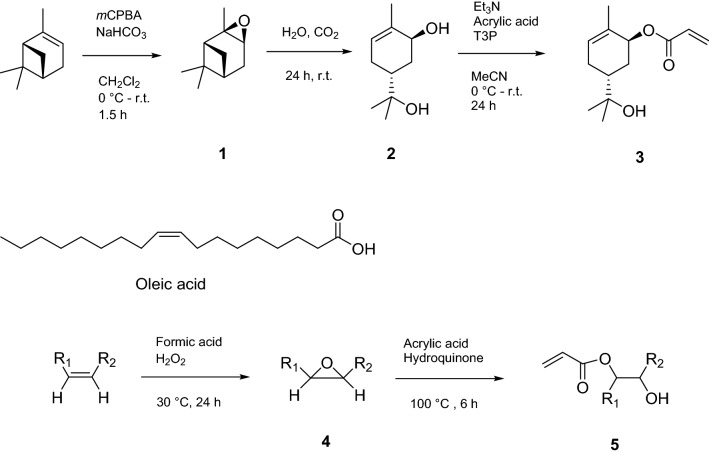
The synthesis routes for the *trans*-sobrerol acrylate monomer (3) and the acrylated oleic acid monomer (5).

The synthesis of the terpene-derived monomer utilised in both polymerisations makes use of α-pinene as the primary starting material (Fig. 1). The first two synthesis steps for this monomer have already been described in previous publications^15,16,21,38^, and consist of first oxidising the terpene (1), followed by its hydrolysation to form *trans*-sobrerol (2). In our previous work^15^, the *trans*-sobrerol was put through a Brown hydroboration/oxidation sequence in order to yield a polyhydroxylated triol molecule. This step was forgone this time, in favour of direct acrylation of *trans*-sobrerol. Although this provides a monomer having one less hydroxyl group, it also makes the process quicker and more scalable. This was an important factor to consider in our work, as the synthesis needed to be efficient enough to be able to make sufficient material for eventual testing on wood, with approximately 50 g of each polymer needed to run a pilot study on wood samples.

The acrylation of *trans*-sobrerol was carried out based on similar reactions previously described^15,16^, with the use of acrylic acid and propanephosphonic acid anhydride (T3P®) as a promoter of the ester coupling. This was deemed preferable to the alternative method which makes use of acryloyl chloride^21^. Unlike the latter reagent, acrylic acid and T3P® produce non-chlorinated waste, making the reaction more environmentally sustainable.

### Monomer synthesis from oleic acid

In addition to the *trans*-sobrerol acrylate monomer (3), we synthesised another monomer in order to create opportunities for the production of copolymers. Oleic acid presented an interesting option as it possesses some attractive properties such as a hydroxylated nature, chain flexibility and ability to create a branched polymeric structure, all of which may potentially improve the ability of its polymer to interact with the wood.

The synthesis of this monomer (Fig. 1) was carried out by following the procedure described by Neto et al*.*^39^ with modifications, and consisted of first epoxidising oleic acid and then using acrylic acid to form the monomer. The epoxidised oleic acid was first obtained by reacting it with formic acid in the presence of hydrogen peroxide. This is possible due to the high reactivity of the unsaturation present in oleic acid, which allows the introduction of functional groups like the epoxide ring^39^. The epoxide ring was then ring-opened with acrylic acid in the presence of hydroquinone. Hydroquinone is a polymerisation inhibitor and is therefore useful in reducing unwanted side reactions, such as acrylic acid polymerisation^39^.

### Thiol-mediated free radical polymerisation of *trans*-sobrerol acrylate (3), TPA6

**Figure 2:**
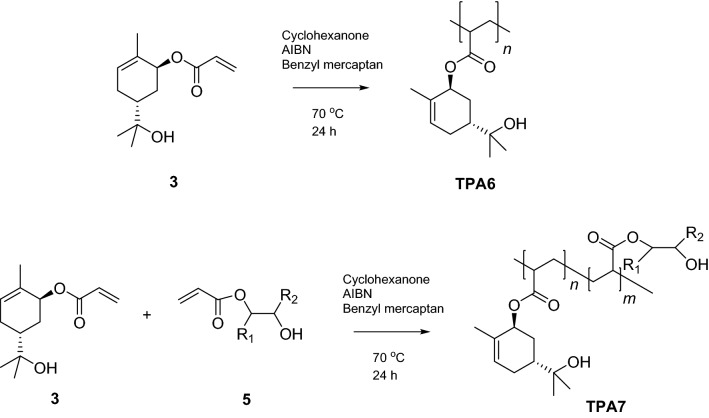
The formation of the *trans*-sobrerol acrylate homopolymer (TPA6) and the *trans*-sobrerol acrylate/acrylated oleic acid copolymer (TPA7).

Free radical polymerisation (FRP) was used for all polymerisation attempts. A molar mass of 5 kDa or below was being targeted. The method previously described for the FRP of sobrerol methacrylate made use of dimethylformamide as the polymerisation solvent and 4,4′-azobis(4-cyanovaleric acid) as the initiator^22^. For our work, the homopolymerisation of *trans*-sobrerol acrylate was first attempted using cyclohexanone as the solvent and azobisisobutyronitrile (AIBN) as the thermal initiator (Fig. 2). Nuclear magnetic resonance (^1^H NMR) analysis indicated that the polymerisation was successful, with the signals representing the acrylate double bond of the monomer no longer appearing in the spectra at the completion of the reaction after purification with hexane (Fig. S7). The gel permeation chromatography (GPC) analysis of this trial FRP reaction indicated that the relative weight average molar mass (*M*_r w_) of this polymer was far too large at 24.3 kDa. It was therefore decided to employ benzyl mercaptan as a chain transfer agent in order to better control its molar mass. Thiols have been employed as efficient, nearly ideal, chain transfer agents thanks to the high efficiency in the control of the chain length attributed to a combination of the weakness of the S–H bond, and the high reactivity of the thiyl radicals (RS·) towards the double bonds^40,41^. A screening was subsequently run on this reaction to identify the optimal conditions (Table 1).

**Table 1:** Homopolymerisation screen of *trans*-sobrerol acrylate (3) as a function of thiol concentration.

Polymer ID	Thiol (mol%)	Starting monomer mass (g)	*M*_r,w_ (kDa)	*M*_r,n_ (kDa)	*Đ*	*T*_g_ (°C)
1	0	0.5	24.3	12.3	2.0	–
2	2	0.5	5.1	3.8	1.4	–
3	8	0.5	3.2	2.3	1.4	–
4	5	0.5	5.0	3.4	1.5	–
5	2	0.5	6.2	4.3	1.5	–
6	5	2.0	3.7	2.8	1.3	–
7	8	2.0	8.7	6.7	1.3	–
8	5	4.0	4.4	3.1	1.4	31.1

*M*_r,w_, *M*_r,n_—relative weight and number average molar masses (relative to poly(methyl methacrylate) standards) from gel permeation chromatography, *Đ* = *M*_r,w_/*M*_r,n._ *T*_g_—glass transition temperature (from DSC).

The first reactions that were carried out with the thiol involved the use of very small amounts of monomer (Entries 2–4 in Table 1). The thiol mol% was varied slightly with each reaction and the molar mass change that it caused was monitored by GPC. A thiol mol% of 2%, 5% and 8% were all tested, all of which produced a polymer with a relative weight average molar mass (relative to polystyrene standards) *M*_r,w_ ranging from 3.2 to 5.1 kDa. Another vital aspect which had to be considered at this point was the reproducibility of the reaction on scale-up. In order to investigate this, a set of reactions with gradually increasing monomer weight were carried out and the resultant polymers’ properties were monitored with GPC. The monomer weight was initially increased to 2.0 g (Entries 7 and 6) and then to 4.0 g (Entry 8). Table 1 demonstrates that the polymers retained similar relative *M*_r,w_, also relative number-average molar mass (*M*_r,n_) and relative dispersity (*Đ*) values when the starting monomer mass was increased from 0.5 to 4.0 g. This suggested that the reaction could be scaled-up further while still retaining the polymer’s characteristics. The *Đ* value is a measure of the different distributions of molecular weight in the system and can have an effect on the polymer’s physical properties. It was therefore important that it remained consistent on scaling-up^42^.

A final thiol amount of 5 mol% was decided upon since it appeared to produce consistent *M*_r,w_ results (Entry 8). It was therefore decided that the conditions used for Entry 8 were the ones that should be utilised for the synthesis of our desired homopolymer, TPA6. The glass transition temperature (*T*_g_) of Entry 8 was thereafter measured with differential scanning calorimetry (DSC). The *T*_g_ is the point at which a polymer changes from a tough or glassy material to a rubbery solid^43^. Having data on the *T*_g_ of a polymer makes it easier for conservators to select consolidants for specific applications^43,44^. The *T*_g_ of TPA6, at the polymerisation conditions selected, was found to be 31.1 °C, which is comparable to that of Paraloid™ B-72 (40.0 °C), a consolidant frequently used in the conservation field^44,45^.

### Copolymerisation of *trans*-sobrerol acrylate (3) and oleic acid acrylate (5), TPA7

Having successfully synthesised the *trans*-sobrerol acrylate monomer (3), the next step was to attempt to copolymerise it with the acrylated oleic acid (5). This would form a copolymer having both terpene- and oleic acid-derived properties. A protocol identical to the one that was used for the homopolymerisation of *trans*-sobrerol acrylate was carried out (Fig. 2). As previously, the reaction was screened in order to decide on the conditions that would be best to produce a polymer with the desired physical properties (Table 2). This involved running several reactions whilst varying the weight (wt) ratios of monomers, along with the amounts of initiator and benzyl mercaptan.

**Table 2:** Copolymerisation screen of *trans*-sobrerol acrylate (3) and oleic acid acrylate (5).

Polymer ID	Monomer 3 (w%)	Monomer 5 (w%)	Thiol (mol %)	AIBN (w%)	*M*_r,w_ (kDa)	*M*_r,n_ (kDa)	*Đ*	*T*_g_ (°C)
1	70	30	0	0.5	16.8	8.2	2.0	–
2	70	30	0	1.0	17.4	7.3	2.4	–
3	30	70	0	1.0	16.4	7.6	2.2	–
4	70	30	0	2.0	17.0	6.9	2.4	–
5	30	70	0	2.0	13.9	5.5	2.5	–
6	70	30	5	1.0	4.2	2.3	1.8	4.6
7	50	50	5	1.0	4.6	2.3	2.0	− 19.0
8	30	70	5	1.0	4.5	2.1	2.1	− 28.0
9	70	30	5	1.0	5.0	2.9	1.8	–
10	80	20	5	1.0	4.0	2.3	1.7	18.9

Monomer ratios shown are the ones that were measured out at the start of each reaction. *M*_r,w_, *M*_r,n_—relative weight and number average molar masses (relative to poly(methyl methacrylate) standards) from gel permetation chromatography. *Đ* = *M*_r,w_/*M*_r,n_.

*T*_*g*_ glass transition temperature (from DSC); w% mass or “weight” %.

A series of reactions (Entry 1–5 in Table 2) was first run without benzyl mercaptan and instead the AIBN concentration was increased from 0.5 to 2.0 wt%. This resulted in the copolymers having a *M*_r,w_ between 13.9 and 17.4 kDa and a *M*_r,n_ between 8.2 and 6.9 kDa. In standard FRP, increasing the thermal initiator concentration would result in more polymer chains likely being initiated, leading to a reduction in molar mass. This is because the Degree of Polymerisation (DP) is inversely proportional to the square root of the initiator concentration^46^. This can be seen from the *M*_r,n_ results in Table 2, with the values decreasing with increasing concentration of thermal initiator.

The addition of 5 mol% of benzyl mercaptan had an immediate effect on all the polymers, with the *M*_r,w_ dropping to between 4.0 and 5.0 kDa. From the DSC analyses of the copolymers we saw that the *T*_g_ decreased with increasing modified oleic acid molar content in the copolymer, since this is closely associated with the final chemical structure of the random copolymer chains. It is reasonable to expect that the plasticising effect of the oleic acid acrylate comonomer influences the molecular mobility of the active species, leading to a reduction in the *T*_g_ of copolymers^47–49^. This property may potentially prove to be beneficial for a wood consolidant as plasticisers are known to increase the free volume of polymer chains, enabling them to be more flexible and move more easily^48,50^. This could possibly translate to the copolymer having more opportunities for interactions with the wood structure once it penetrates, although its plasticity should not be too high as to deform the wood. Ideally, the *T*_g_ of a consolidant should be higher than the maximum temperature that a treated artefact will be subjected to^51^. A *T*_g_ which is too low may result in slumping of the object, as has been reported happening with the use of Paraloid™ B-72 in hot climates^44,52,53^. On the other hand, a consolidant which is too stiff may cause artefacts to become brittle, which can result in cracking if put under stress^54^. Keeping this in mind, it was decided that the best conditions to use for TPA7 would be those utilised for Entry 10 in Table 2, as the copolymer had a small enough *M*_r,w_ (4.0 kDa) as well as the most appropriate *T*_g_ (18.9 °C).

### Hydrodynamic characterisation studies of TPA6 and TPA7

Having a thorough understanding of the physical properties of TPA6 and TPA7 is vital in order to help us in deciding whether they would truly be suitable as wood consolidants. It is essential to have as much certainty as possible since any scale-up of the polymers would entail a considerable amount of work and time. Moreover, archaeological wood which can be used for any subsequent testing is scarce and thus, extensive characterisation of the polymers would help in preventing material wastage.

As mentioned, analytical ultracentrifugation (AUC) is matrix-free—not requiring a separation matrix or membrane and works without the need of calibration standards i.e. it is an absolute method of estimating molecular weight^32^. This is in contrast to the relative method of gel permeation chromatography or GPC, which compares the tested polymers to built-in standards such as poly(methyl methacrylate) standards. As a result, AUC is considered to be a more reliable method and the molar mass values that are obtained from it are more accurate than the ones measured by GPC, as they are absolute and not dependent on standards, and without assumptions of column inertness. Because of this, once the polymerisation reactions of TPA6 and TPA7 were optimised, it was decided to re-measure their molecular weights with AUC in order to obtain more accurate or absolute values.

AUC also helps in the determination of molecular weight distribution and conformation of the polymers. This is useful for wood consolidation purposes, since it would help to predict how much of the polymer fraction would be able to penetrate wood. If a major polymer fraction is deemed too large (approximately > 10 kDa), it would not be scientifically sound to continue with their scale-up and testing^55^. Likewise, having an idea of the shape of the polymers though conformation analysis could also help in understanding their behaviour. An example is PEG, whose long and flexible shape might improve its penetration into wood^55^.

### Calculation of the partial specific volume $\overline{\nu }$

**Figure 3:**
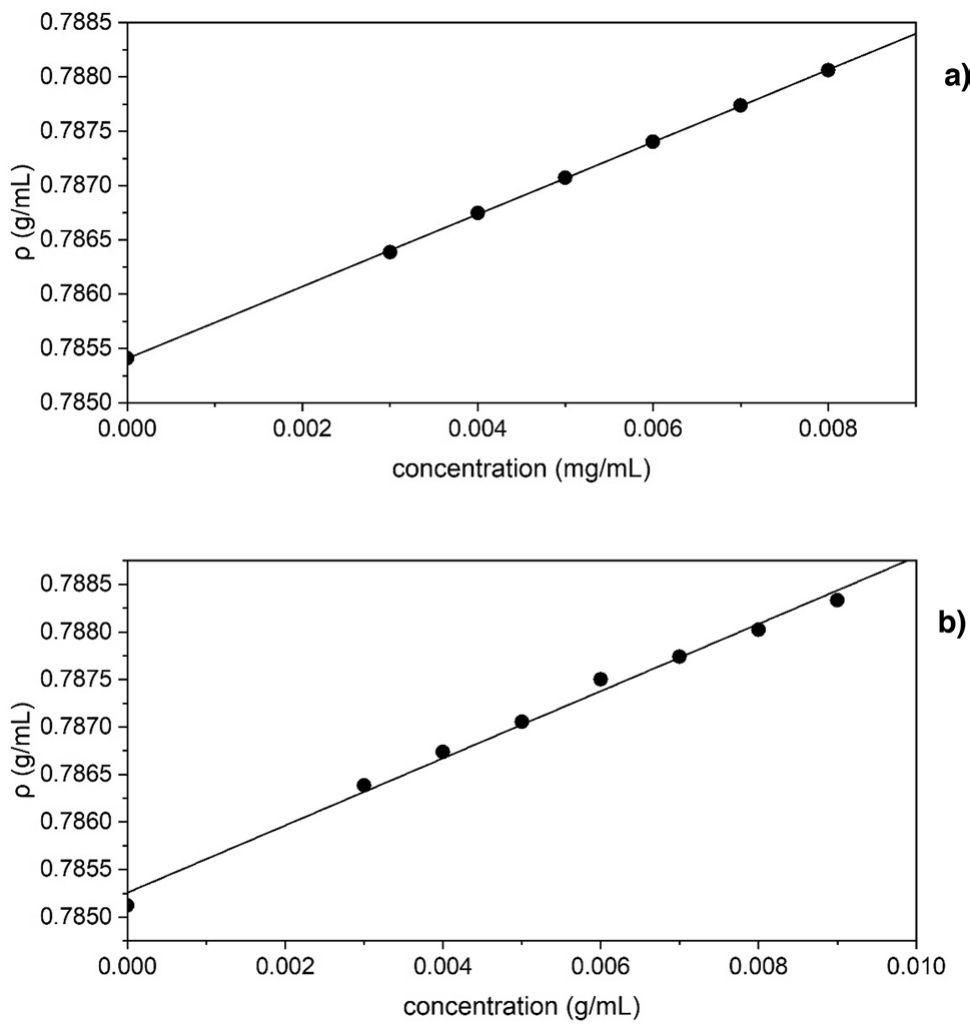
Dependence of solution density of TPA6 (**a**) and TPA7 (**b**) in isopropanol on concentration.

The partial specific volumes $\overline{\nu }$ of both polymers was measured since these values are essential for the analyses of the subsequent AUC studies. The method previously described by Kratky et al*.*^56^ was followed. This involved measuring the density of different concentrations of polymer dissolved in an appropriate solvent. It was decided to carry out all characterisation studies in isopropanol since it has been previously used in experiments with degraded alum-treated wood^57^. These density values were then plotted against concentration (Fig. 3) and Eq. (1) was used to obtain the $\overline{\nu }$ values. These were found to be (0.850 ± 0.002) and (0.824 ± 0.013) cm^3^/g for TPA6 and TPA7 respectively. These were similar to the value that had previously been measured for our other terpene-derived acrylated polymer^15^.

1 $$\overline{v} = \frac{1}{{\rho_{o} }}\left( {1 - \frac{\partial \rho }{{\partial c}}} \right)$$

where *ρ*_o_—the density of the reference solvent; $\frac{\partial \rho }{\partial c}$—the slope of the *ρ* versus concentration.

### Sedimentation velocity in the analytical ultracentrifuge

**Figure 4:**
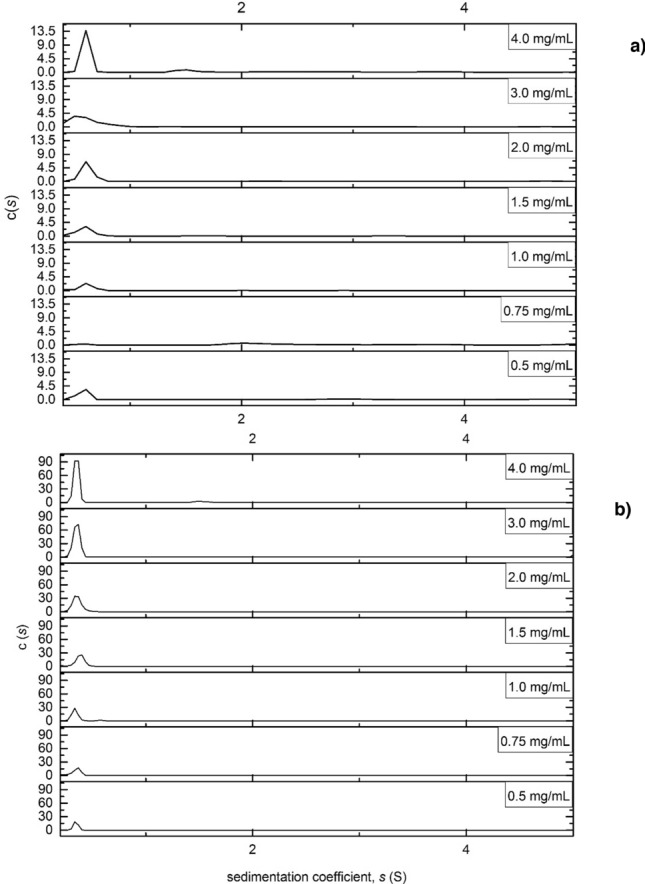
Sedimentation coefficient distributions c(*s*) vs sedimentation coefficient (S) for (**a**) TPA6 and (**b**) TPA7 in isopropanol. Rotor speed = 49,000 rpm.

Sedimentation velocity (SV) studies were carried out on both polymers in isopropanol in order to get an indication of their degree of heterogeneity. SEDFIT analyses provided us with the sedimentation coefficient range *c(s)*^58^ vs sedimentation coefficient (S) of the polymers (Fig. 4). Both polymer systems appeared to be similar, with the major peak having a low sedimentation coefficient value (ca. 0.6 and 0.4 S for TPA6 and TPA7 respectively), which corresponds with the particle having a small *M*_w_ value. Some minor peaks were observed at ~1.5 S, indicating that the polymer systems were also composed of some larger *M*_w_ particles. These were more pronounced in TPA6, which could potentially mean that this polymer may have a tendency to aggregate at higher concentrations, especially above 1.5 mg/mL.

### Sedimentation equilibrium in the analytical ultracentrifuge

**Figure 5:**
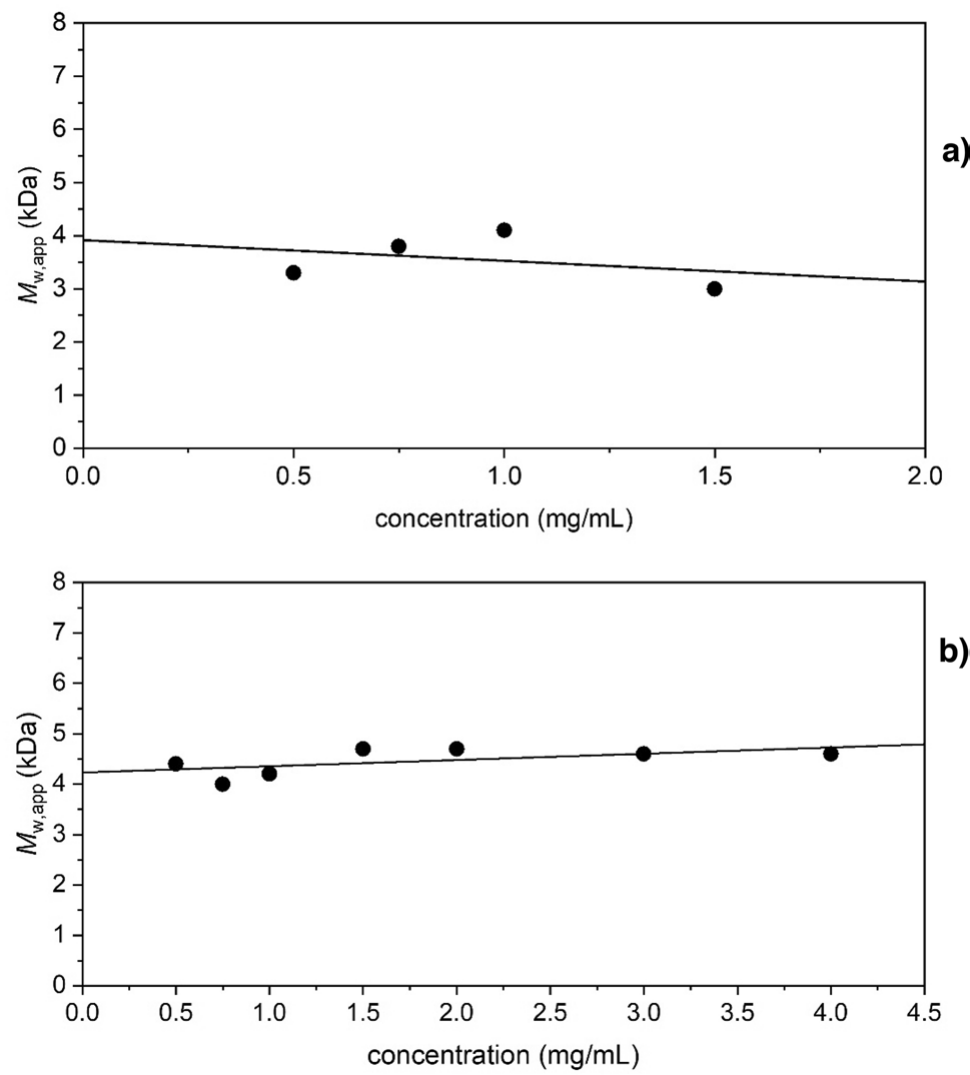
Dependence of apparent *M*_*w,app*_ on concentration, with an extrapolation to obtain the thermodynamically ideal *M*_*w,app*_. Analysed with SEDFIT-MSTAR using the M* derived method to obtain the shown *M*_*w,app*_ values. Rotor speed = 45,000 rpm. (**a**) TPA6 in isopropanol, *M*_w_ = (3.9 ± 0.8) kDa; (**b**) TPA7 in isopropanol, *M*_w_ = (4.2 ± 0.2) kDa.

Sedimentation equilibrium (SE) experiments were run on both polymers, using a concentration series ranging from 0.5 to 4.0 mg/mL, to estimate absolute molar masses. As stressed previously although not dependent on poly(methyl methacrylate) standards like GPC which are assumed to have the same conformation, nonetheless sedimentation equilibrium yields *apparent* molar masses (i.e. affected by non-ideality through polymer co-exclusion) *M*_app_. Using different concentrations of polymer allowed us to determine the extent of the polymers’ non-ideal behaviour, and either a simple extrapolation to zero concentration (or operating at a low concentration) eliminates these effects. SEDFIT-MSTAR^59^ was used to obtain the apparent weight average molar mass *M*_w,app_ via the M* function^60^ and the hinge-point method^59^. Tables S1 and S2 (Supplementary Information) show these values. From this data we could see that there seems to be relatively low non-ideality in both polymer systems, as the *M*_w,app_ did not appear to be significantly affected by the concentration. This was not surprising for polymers with such low molar masses. For TPA6 it was decided not to use polymer concentrations above 1.5 mg/mL, due to the possibility of aggregation as was noted during the SV study. The “ideal” average *M*_w_ values were then determined by plotting the *M*_w,app_ against concentration (Fig. 5) and extrapolating to zero concentration. These were found to be (3.9 ± 0.8) kDa and (4.2 ± 0.2) kDa for TPA6 and TPA7 respectively.

**Figure 6:**
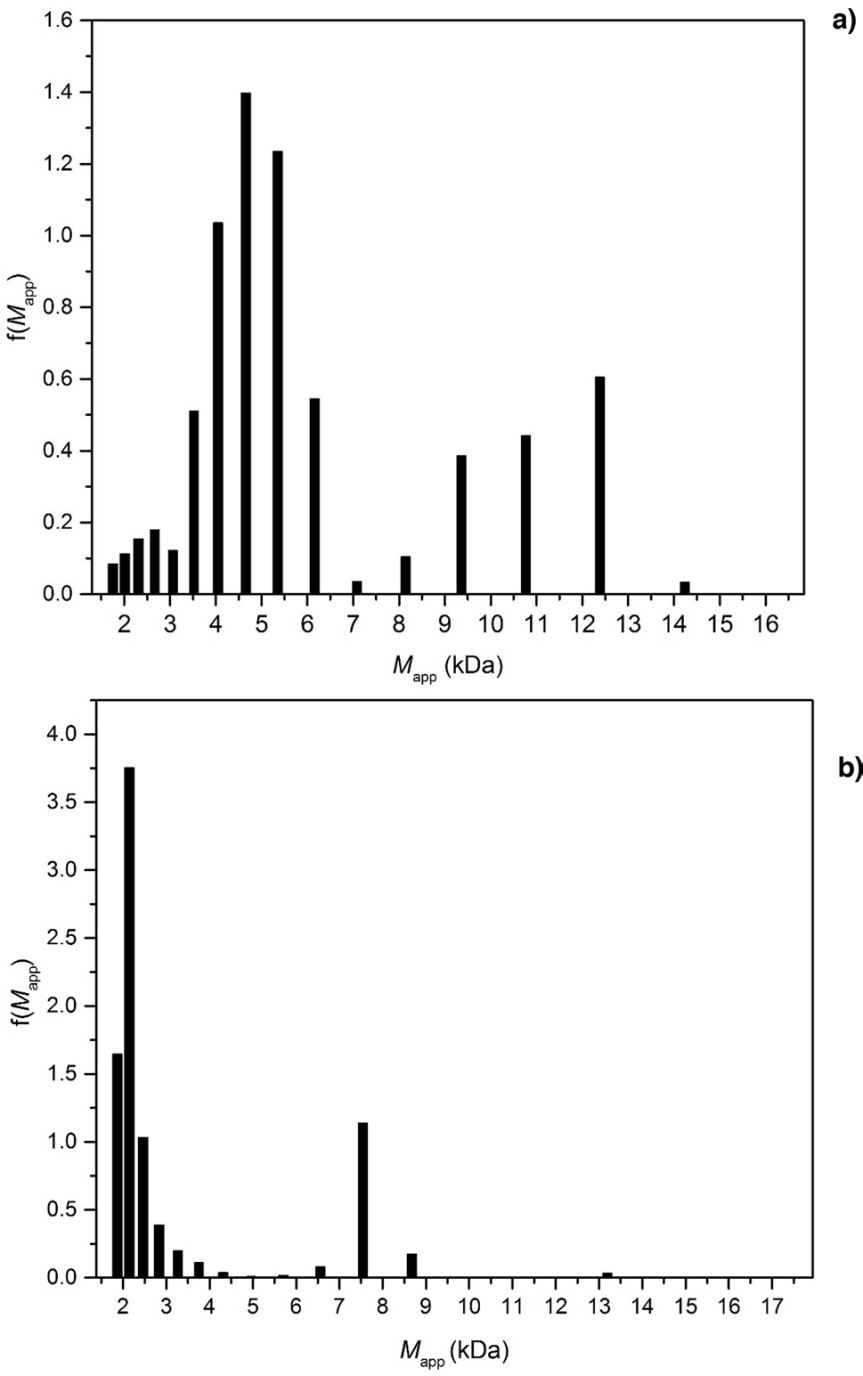
MultiSig analyses of the molar mass distribution f(*M*) vs *M*_w_ of (**a**) TPA6 and (**b**) TPA7 at a loading concentration of 4.0 mg/mL. Rotational speed = 45,000 rpm.

The SE data was also run through the MultiSig programme, which provides an estimate of the molar mass *distribution*, assuming ideal behaviour^61^. Figure 6a shows the results for TPA6, indicating that it had an *M*_w_ range from 1.8 to 14.5 kDa, with a main peak at 4.7 kDa, but a 2nd significant and broad peak from 7 to 14.5 kDa. The results for TPA7 are shown in Fig. 6b, which reveals that the *M*_w_ distribution of this polymer system was considerably narrower ranging from 1.9 to 4.4 kDa, with a smaller peak at ~ 7.6 kDa. The results for both polymers appeared to correlate with the ones that were obtained from the SV data, showing that the majority of particles in the polymer systems had a low *M*_w_ (and in the targeted range for a consolidant) and with TPA6 being considerably more polydisperse.

### Calculation of the intrinsic viscosity $\left[ {\varvec{\eta}} \right]$

A rolling ball viscometer was used to measure the viscosity of both polymers in isopropanol. This instrument was specifically chosen since it carries out the measurements in a closed environment, thereby minimising the degree of evaporation of the highly volatile isopropanol. With these measurements, the Solomon–Ciuta equation (Eq. (2)) was then implemented to calculate the final $\left[\eta \right]$ value for the polymers. These were (4.88 ± 0.24) and (4.84 ± 0.24) mL/g for TPA6 and TPA7 respectively.

2 $$\left[\eta \right]= \frac{1}{c}{\left(2\left({\eta }_{sp}\right)-2\mathrm{ln}({\eta }_{r})\right)}^\frac{1}{2}$$

*c* = concentration of polymer solution; $\eta$ = solution viscosity; ${\eta }_{0}$ = viscosity of pure solvent; ${\eta }_{r}$= relative viscosity ($\frac{\eta }{{\eta }_{0}}$); ${\eta }_{sp}$= specific viscosity (${\eta }_{r}$ − 1).

### Conformation analyses

With the values for the $\overline{\nu }$, *M*_w_, and $\left[\eta \right]$ obtained from previous experiments, the conformation of the polymers could then be investigated. This is particularly important as the shape of a polymer in a particular solvent may help us understand its behaviour once it is absorbed by the wood.

**Figure 7:**
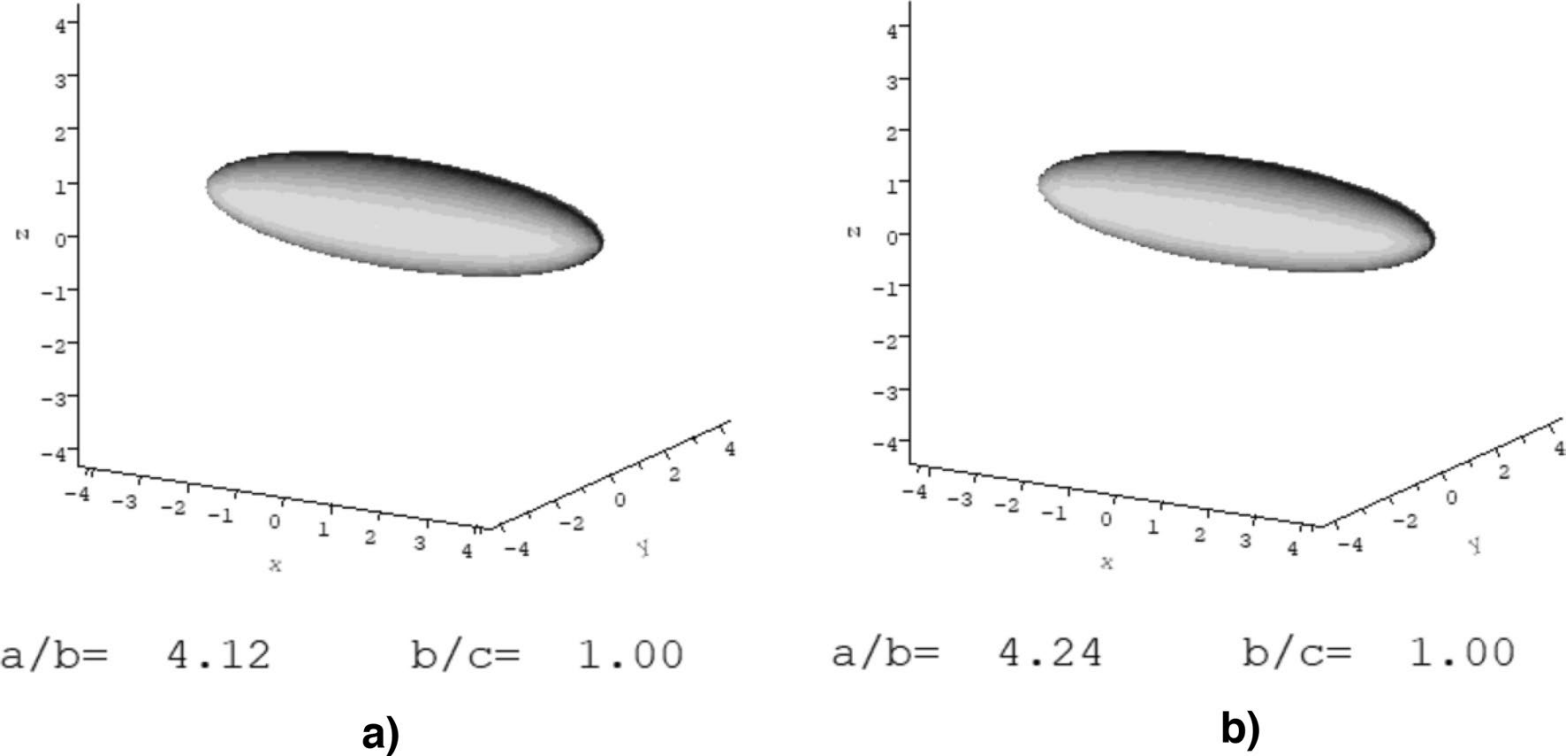
Ellipsoidal representations from the conformation analysis of (**a**) TPA6 and (**b**) TPA7 in isopropanol, using the programme ELLIPS1 with the shape parameter $\upnu$. The polymers, within experimental error, have identical axial ratios.

The viscosity increment ($\upnu$) factor was used to predict the axial ratio of TPA6 and TPA7 with the programme ELLIPS1^62^. $\upnu$, described by Eq. (3)^63–66^, is a universal shape parameter meaning that it is only affected by the particle’s shape and is independent of its size^67^.

3 $$\upnu = \left[\eta \right]/{v}_{s}$$

where ${v}_{s}$ is the swollen (through solvent association) specific volume of the polymer (mL/g). ELLIPS1 works by converting the value for a shape function to an estimate of the axial ratio $(a/b)$ for ellipsoids of revolution^62^. Table 3 shows the values for the $\upnu$ parameter and the corresponding axial ratios for both polymers at different degrees of solvent association, which is the degree of interaction between a polymer in solution and a solvent (defined as $\nu_{s} /\overline{\nu }$, where ${v}_{s}$ is the swollen specific volume and $\overline{\nu }$ is the partial specific volume). If the polymers did not bind with isopropanol, then the solvent association would be equal to their $\overline{\nu }$. In Table 3, solvent association values of 1.0–1.4 were used for the shape analyses in order to determine whether these would drastically affect the results. Figure 7 shows the visual representation of the shapes (prolate ellipsoid model) of the polymers estimated from these values and indicates that both TPA6 and TPA7 appear to have a long, oval shape. This property may prove to be advantageous for consolidants, as this elongated shape may potentially increase the surface area available for possible interactions between the wood and the polymers.

**Table 3:** The calculated values for the shape parameter $\upnu$ and the axial ratios (prolate ellipsoid model) determined by ELLIPS1.

		Degree of solvent association $\nu_{s} /\overline{\nu }$		
		1	1.2	1.4
TPA6	Shape factor $\upnu$	5.7 ± 0.6	4.8 ± 0.6	4.1 ± 0.6
	Axial ratio $(a/b)$	5.0	4.1	3.4
TPA7	Shape factor $\upnu$	5.7 ± 0.3	4.9 ± 0.3	4.2 ± 0.3
	Axial ratio $(a/b)$	4.9	4.2	3.6

## Conclusion

Two polymers have successfully been synthesised, from α-pinene- and oleic acid-derived monomers. Both monomers can easily undergo FRP and their feedstocks are cheap and highly accessible. Moreover, the other reagents that are utilised in the syntheses are commonly used products which are available commercially. A new protocol for the polymerisation of *trans*-sobrerol acrylate has been described, and a new copolymer composed of *trans*-sobrerol acrylate and acrylated oleic acid has been successfully synthesised. The conditions for the polymerisations appear to be easily reproducible even on scale-up, which would be very beneficial for the eventual testing on wood and for their practical use as conservation materials. Bioinspired polymers such as TPA6 and TPA7 have reduced negative impact on the environment by virtue of being sourced from biomass, however their production cannot be considered to be truly sustainable^7^. Every effort has been made to make the chemical synthesis processes as ‘green’ as possible, these included using water and mild conditions (the synthesis of *trans*-sobrerol) and making reagent substitutions when appropriate (replacing acryloyl chloride with acrylic acid and T3P®). It should be recognised however that these processes and reagents are still far from being environmentally harmless. More optimisation of the monomer and polymer synthesis protocols can therefore be carried out in the future to attempt to make them more sustainable.

The hydrodynamic characterisation analyses indicated that both polymers have good properties for wood consolidation such as a *M*_w_ below 5 kDa and an elongated shape but with TPA7 showing greater homogeneity and a lower proportion of aggregation. These are all advantageous properties for a wood consolidant to have, indicating that these two polymers should be investigated further. It should be noted that these studies are still preliminary and that it would be essential for further investigations to be carried out before TPA6 and TPA7 can be considered as official candidates for wood consolidation. This would entail first confirming whether they can penetrate archaeological wood and analysing their consolidative behaviour. Should this prove successful, long-term stability studies will be essential as these would provide information on how these polymers fare in the wood after months or years. In order to be a viable consolidant, more information would also be needed on their toxicity and by-product formation^7,55^.

As such, the next step for this research will involve testing these polymers on archaeological wood in order to determine whether they can sufficiently penetrate it and to observe the effects that they have on it^55^. This will involve dissolving the polymers in a suitable solvent (such as isopropanol) and then immersing a number of archaeological wood samples in the solutions for an appropriate length of time. After drying, these samples can then be studied to determine the effect of the polymers on the wood. This would involve analysing the samples with infrared spectroscopy and SEM to confirm the penetration of the polymers^55^. Weight change, dimensional change and colour change can also be monitored before and after treatment. The consolidative effect of the polymers can be investigated by testing the hardness of the treated wood, for example with the use of penetrometery which measures resistance to indentation^55^. These studies will give us essential information on the potential of these bioinspired compounds as wood consolidants, and whether they can successfully be used as part of the “polymer treatment toolkit” for the Oseberg artefacts and other archaeological wooden objects under threat in the future.

## Materials and methods

### Materials

All reagents and solvents were purchased from a chemical supplier (Acros Organics, Alfa Aesar, Merck, Sigma Aldrich and Fischer Scientific UK) and used without further purification. Water was deionised before use. Brine is a saturated aqueous solution of sodium chloride. Rotary evaporators under reduced pressure were used for solvent evaporation.

### Nuclear magnetic resonance (NMR)

^1^H NMR spectra were recorded in deuterated chloroform (CDCl_3_), deuterated DMSO ((CD_3_)_2_SO) and deuterated methanol (CD_3_OD) at ambient temperature using Bruker 400 MHz spectrometers (Bruker Corporation, Germany). ^13^C NMR spectra were recorded in CDCl_3_, (CD_3_)_2_SO and CD_3_OD at ambient temperature using 100 MHz spectrometers. Data is expressed as chemical shifts (δ) in ppm relative to solvent signals (C*H*Cl_3_, ^1^H NMR 7.26), (*C*DCl_3_^13^C NMR 77.16), ((C*H*_3_)_2_SO, ^1^H NMR 2.50), ((*C*D_3_)_2_SO, ^13^C NMR 39.52), (C*H*_3_OH, ^1^H NMR 3.31) or (*C*D_3_OD, ^13^C NMR 49.0) as the internal standard. MestReNova 6.0.2 copyright 2009 (Mestrelab Research S.L.) was used for analysing the spectra. The data is available in the Supplementary Information.

### High resolution mass spectrometry (HRMS)

A Bruker MicroTOF spectrometer operating in electrospray ionisation (ESI) mode was used (Bruker Corporation, Germany).

### Fourier-transform infra-red spectroscopy (FTIR)

A Bruker Tensor 27 FT-IR spectrophotometer with an ATR attachment was employed. The measurements were performed in the range of 4000–650 cm^−1^ and spectra were analysed using OPUS software (Bruker Corporation, Germany).

### Gel permeation chromatography (GPC)

An Agilent 1260 Infinity Series HPLC (Agilent Technologies, USA) fitted with a differential refractive index detector (DRI) was used. THF (HPLC grade, Fisher Scientific) was used as the eluent at room temperature with two Agilent PL-gel mixed-D columns in series at a flow rate of 1 mL/min. A calibration curve was made using poly(methyl methacrylate) standards with ASTRA software (Wyatt Technology, USA) This was used for determination of the relative (compared to the standards) *M*_r,n_, *M*_r,w_ and molar mass distribution and dispersity (*Đ* = *M*_r,w_/*M*_r,n_).

### Differential scanning calorimetry (DSC)

A TA-Q2000 (TA instruments) calibrated with an indium standard under an N_2_ flow was used to measure *T*_g_. A The sample (~ 5 mg) was weighed in a T-zero sample pan (TA instruments), leaving another T-zero pan empty as a reference. Both pans were heated at a rate of 10 °C/min, using a heat/cool/heat method.

### Monomer and polymer synthesis

The synthesis of α-pinene oxide (1) and *trans*-sobrerol (2) has been reported in a previous publication^15^. The epoxidised oleic acid (4) and the acrylated oleic acid (5) were synthesised following the protocols described by Neto et al.^39^. The Supplementary Information contains details on these methodologies.

#### Synthesis of trans-sobrerol acrylate, 3

To a solution of **2** (7.08 g, 41.6 mmol) in MeCN (200 mL) were added Et_3_N (17.5 mL, 125.6 mmol), acrylic acid (acid with low H_2_O content, 99.5% stab. with ca. 200 ppm methoxyphenol, 3.14 mL, 45.8 mmol) and then T3P® (50 wt% in ethyl acetate, 29.7 mL, 99.8 mmol) was added dropwise. The reaction mixture was stirred for 24 h, after which H_2_O (200 mL) was added and the reaction mixture separated. The aqueous layer was separated with diethyl ether (100 mL × 3) and the combined organic layers were washed with HCl (1 M aqueous, 200 mL × 3), NaHCO_3_ (sat. aq., 200 mL × 3) and brine (100 mL × 2). The reaction mixture was then dried with MgSO_4_, filtered and concentrated to yield the title compound (**3**) as a brownish orange viscous liquid (6.05 g, 27.0 mmol, 65% yield).

#### General procedure for the polymerisation of trans-sobrerol acrylate, 3, to yield a terpene-derived acrylated polymer

##### TPA6

To the monomer (4 g, 17.8 mmol) was added cyclohexanone (24 mL), AIBN (0.5 wt%, 20 mg, 0.1 mmol) and benzyl mercaptan (5 mol%, 112 µL, 1 mmol). The mixture was purged with argon for 1 h 45 min, after which it was stirred for 24 h at 70.0 °C. The mixture was left to cool to room temperature and then purified with excess of hexane (4:1 v/v). The product was dried in a room temperature vacuum oven to yield the title compound (TPA6).

#### General procedure for the copolymerisation of trans-sobrerol acrylate 3 and acrylated oleic acid 5, to yield a terpene- and oleic acid-derived acrylated copolymer

##### TPA7

To a mixture of monomer 3 (6.4 g, 28.5 mmol) and monomer 5 (1.6 g, 4.3 mmol) was added cyclohexanone (48 mL), AIBN (1 wt%, 80 mg, 0.5 mmol) and benzyl mercaptan (5 mol%, 223.9 µL, 2 mmol). The mixture was purged with argon for 1 h 45 min, after which it was stirred for 24 h at 70.0 °C. The mixture was left to cool to room temperature and then purified with excess of hexane (4:1 v/v). The product was dried in a room temperature vacuum oven to yield the title compound (TPA7).

### Density measurements: calculation of the partial specific volume ($\overline{\nu }$)

An Anton Paar DMA 5000 V5.003 was used at 20.0 °C. The partial specific volume is required for the analyses of the sedimentation velocity, sedimentation equilibrium studies and conformational analyses. A 9.0 mg/mL stock solution of each TPA6 and TPA7 in isopropanol were prepared and then diluted to 8.0, 7.0, 6.0, 5.0 and 4.0 mg/mL. These concentrations were thereafter used for the density measurements. The results were evaluated using Eq. (1) and following the procedure of Kratky et al.^56^.

### Analytical ultracentrifugation (AUC)

A Beckman Optima XL-I analytical ultracentrifuge with Rayleigh interference optics was used at 20.0 °C. 12 mm optical path length double sector cells with titanium centrepieces were employed.

#### Sedimentation velocity

Loading concentrations of 0.5 to 4.0 mg/mL of TPA6 and TPA7 in isopropanol were used. 405 μL of each concentration were injected in the sample solution channel of each AUC cell. Isopropanol was used as the reference solution. A rotational speed of 49,000 rpm was used and the samples centrifuged overnight. The weight average sedimentation coefficient (*s*) and the distributions of sedimentation coefficient *c(s)* vs s were obtained by analysis with the SEDFIT procedure^68^. This analysis was carried out to assess the size distribution and heterogeneity of the polymer systems.

#### Sedimentation equilibrium

100 μL of the previous loading concentrations (0.5 to 4.0 mg/mL) of TPA6 and TPA7 in isopropanol were added to each of the AUC cells. Isopropanol was used as the reference solution. The experiment was carried out at a rotational speed of 45,000 rpm over 2 days. The results were analysed with SEDFIT-MSTAR^59^ in order to obtain the apparent weight average molar mass (*M*_w,app_), making use of the M* extrapolation^60^ and the hinge point method. No major concentration dependence was observed for either polymer systems, which suggested that non-ideality was not significant. The data obtained from the highest concentration (4.0 mg/mL) of both polymers was additionally analysed with the MultiSig algorithm^61^ to evaluate the extent of any aggregation.

### Viscosity measurements: calculation of the intrinsic viscosity [η]

An Anton-Paar AMVn (Graz, Austria) rolling ball viscometer was used at a temperature of 10.0 °C. This low temperature was chosen so as to prevent the solvent (isopropanol) from evaporating due to its high volatility.

The viscosity measurement was carried out using solutions of 6.0 mg/mL concentration of TPA6 and TPA7 in isopropanol. The intrinsic viscosity values were then calculated with the Solomon-Ciuta equation^69,70^ (Eq. (2)).

### Conformation analyses

The ELLIPS1^62^ algorithm was used to estimate macromolecular asymmetry from the viscosity increment ($\upnu$) using Eq. (3).

## Supplementary Information

Supplementary Information.

## Data Availability

Raw data is available in Supplementary Information. Additional information is available from the Corresponding Authors.
