# Supplementary material for: Comparative hydrodynamic characterisation of two hydroxylated polymers based on α-pinene- or oleic acid-derived monomers for potential use as archaeological consolidants
Source: Sci Rep. 2022 Nov 1;12:18411. doi: 10.1038/s41598-022-21027-4 (PMC9626589; doi:10.1038/s41598-022-21027-4)
Supplement: Supplementary file 1 — Supplementary Information. [file 41598_2022_21027_MOESM1_ESM.docx]

**Supplementary Material**

**Comparative hydrodynamic characterisation of two hydroxylated polymers based on α-pinene- or oleic acid-derived monomers for potential use as archaeological consolidants**

Michelle Cutajar^1,2*^, Fabricio Machado^2,3^, Valentina Cuzzucoli Crucitti^4^, Susan Braovac^5^, Robert A. Stockman^2^, Steven M. Howdle^2^ and Stephen E. Harding^1,5*^

1. *National Centre for Macromolecular Hydrodynamics (NCMH), University of Nottingham, School of Biosciences, Sutton Bonington, LE12 5RD, U**K*
2. *School of Chemistry, University of Nottingham, University Park Nottingham, NG7 2RD UK*
3. *Instituto de Química, Universidade de Brasília, Campus Universitário Darcy Ribeiro, 70910-900 Brasília, DF, Brazil*
4. *Centre for Additive Manufacturing, Department of Chemical and Environmental Engineering, Faculty of Engineering, University of Nottingham, Nottingham, NG7 2RD UK*
5. *Museum of Cultural History, University of Oslo, Kabelgata 34, 0580 Oslo, Norway*

**α-Pinene oxide (1)**

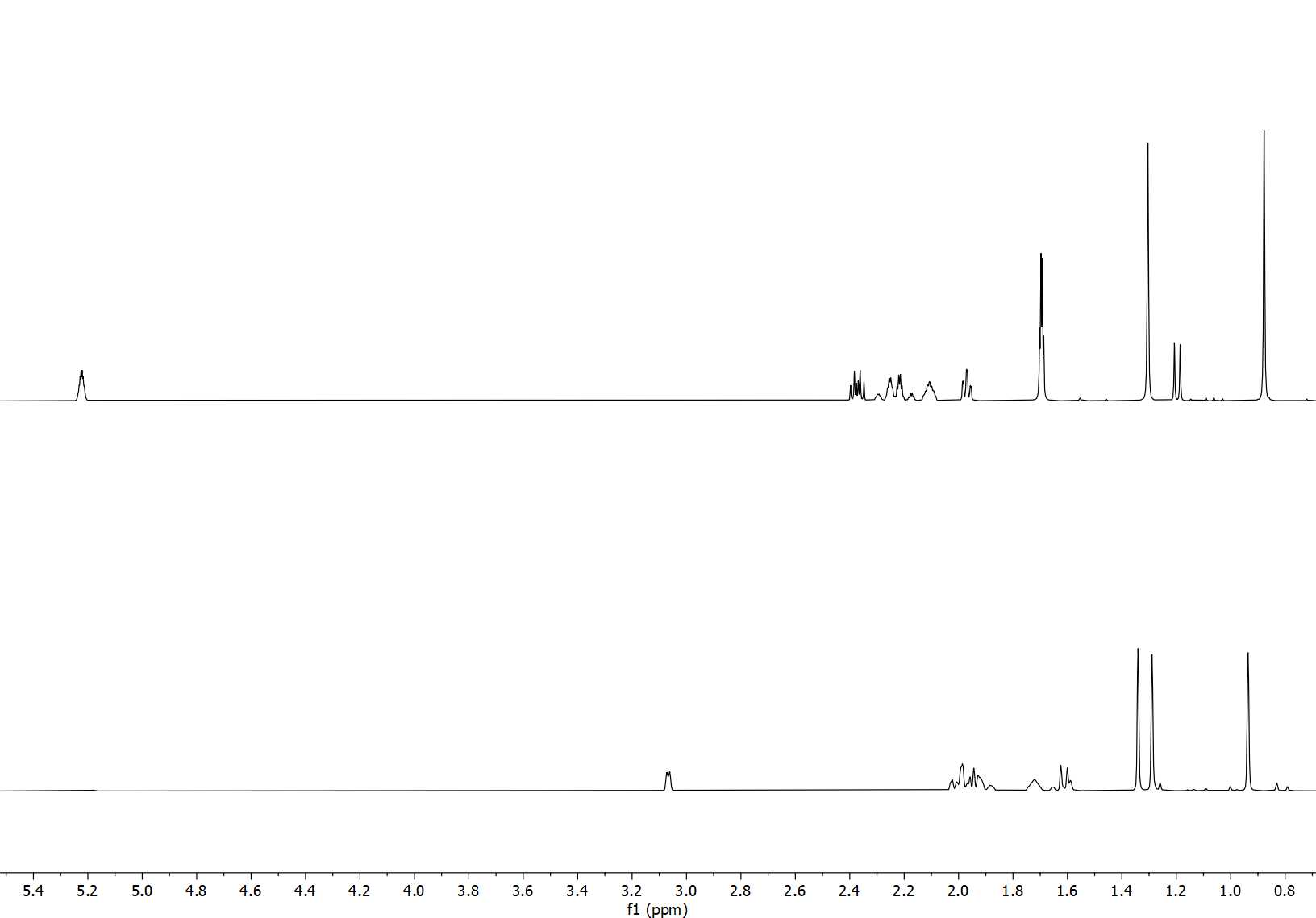


**Figure S1.** ^1^H NMR spectra for **1**

*Synthesis of α-pinene oxide,* ***1***^1^*.* 1S-(-)-α-pinene (5.82 mL, 36.7 mmol) was added to a suspension of NaHCO_3_ (3.92 g, 46.6 mmol) in CH_2_Cl_2_ (7.5 mL) and then cooled to 0 °C. *Meta*-chloroperbenzoic acid (*m*CPBA) (~70%, 9.22 g, 37.4 mmol) was gradually added to the solution. The reaction was stirred for 1 hour, after which saturated aqueous solution of Na_2_SO_3_ (27 mL) was added to the reaction mixture. The reaction was allowed to settle to room temperature and stirred for a further 30 minutes. The reaction mixture was diluted with saturated aqueous solution of NaHCO_3_ (30 mL) and CH_2_Cl_2_ (60 mL). The aqueous washings were extracted with CH_2_Cl_2_ (75 mL). The organic phase was washed with saturated aqueous solution of NaHCO_3_ (100 mL x 3). The organic extracts were then combined, washed with brine (100 mL x 3), dried over MgSO_4_, filtered and concentrated under reduced pressure to yield the title compound (**1**) (4.78 g, 31.4 mmol, 86% yield).

**FTIR** (ATR) ν_max_ /cm^-1^: 2977, 2914, 2834, 1229, 1084, 943, 818; **^1^H NMR** (400 MHz, CDCl_3_) δ_H_ 3.07 (d, *J* = 4.1 Hz, 1H), 2.01 – 1.83 (m, 4H), 1.72 (s, 1H), 1.61 (d, *J* = 9.4, 1H), 1.34 (s, 3H), 1.29 (s, 3H), 0.94 (s, 3H); **^13^C NMR** (100 MHz, CDCl_3_) δ_C_ 60.3, 56.9, 45.1, 40.5, 39.7, 27.6, 26.7, 25.9, 22.4, 20.2.

***Trans*-sobrerol (2)**

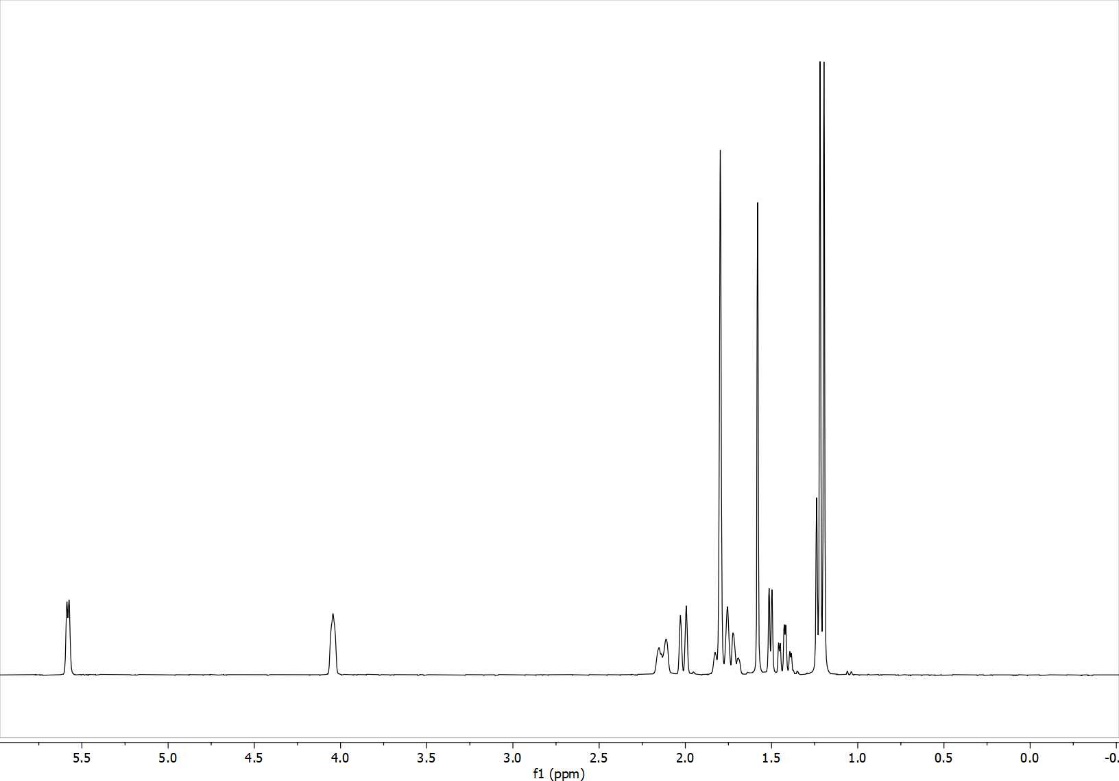


**Figure S2.** ^1^H NMR spectra for **2**

*Synthesis of trans-sobrerol,* ***2****.*^1^ CO_2_ was continuously passed through H_2_O (52 mL) until the pH was approximately 4.5 – 5. **1** (4 g, 26.3 mmol) was then added and the mixture stirred at room temperature for 24 hours. The solution was concentrated under reduced pressure and a white solid precipitated. The crude solid was washed with cold ethyl acetate (5 mL x 2) to give the title compound as a white, crystalline solid (**2**) (2.4 g, 14.4 mmol, 55% yield).

**FTIR** (ATR) ν_max_ /cm^-1^: 3321, 2973, 2887, 1376, 1052, 919; **^1^H NMR** (400 MHz, CDCl_3_) δ_H_ 5.58 (d, *J* = 5.4 Hz, 1H), 4.04 (s, 1H), 2.17 – 2.08 (m, 1H), 2.05 – 1.97 (m, 1H), 1.84 – 1.67 (m, 5H), 1.42 (td, *J* = 13.1, 3.9 Hz, 1H), 1.22 (s, 3H), 1.19 (s, 3H); **^13^C NMR** (100 MHz, CDCl_3_) δ_C_ 133.2, 126.6, 71.3, 68.8, 38.9, 33.8, 27.8, 27.3, 26.5, 21.0; **HRMS** (ESI) m/z calculated for [C_10_H_18_NaO_2_]^+^ 193.1204 found 193.1210 (M^+^ Na^+^).

**(1S,5R)-5-(2-hydroxypropan-2-yl)-2-methylcyclohex-2-en-1-yl acrylate (3)**

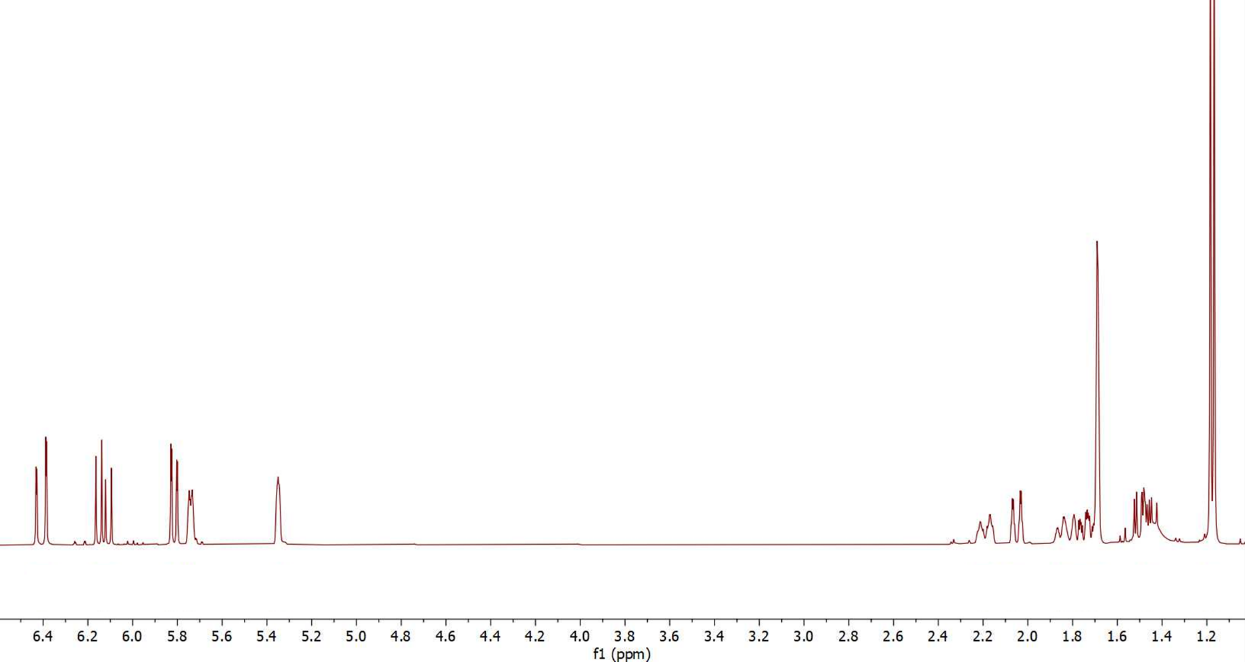


**Figure S3.** ^1^H NMR spectra for **3**

**FTIR** (ATR) ν_max_ /cm^-1^: 3421, 2969, 2935, 1717, 1704, 1404, 1294, 1267, 1192, 1162, 1038. **^1^H NMR** (400 MHz, CD_3_OD) δ_H_ 6.42 (dd, *J* = 17.3, 1.5, 1H), 6.14 (dd, *J* = 17.3, 10.4, 1H, H-12), 5.82 (dd, *J* = 10.4, 1.5, 1H), 5.75 (dt, *J* = 5.6, 1.8, 1H), 5.36 (dt, *J* = 3.5, 1H), 2.19 (dddt, *J* = 17.0, 5.7, 4.3, 1.6, 1H), 2.05 (dq, *J* = 14.0, 2.2, 1H), 1.90–1.82 (m, 1H), 1.74 (tdd, *J* = 2.4, 4.0, 12.5, 1H), 1.71 (dt, *J* = 2.8, 1.5, 3H), 1.49 (ddd, *J* = 14.1, 12.9, 4.0, 2H, H-6), 1.18 (*J* = 6.9, 6H); **^13^C NMR** (101 MHz, CD_3_OD) δ_C_ 166.1, 131.0, 130.6, 128.1, 125.3, 72.2, 71.0, 39.5, 30.0, 27.6, 27.4, 26.8, 20.9; **HRMS** (ESI): Calculated for [C_13_H_20_NaO_3_]^+^ 247.3000 obtained 247.1309 (M^+^ Na^+^).

**8-(3-octyloxiran-2-yl)octanoic acid (4)**

**
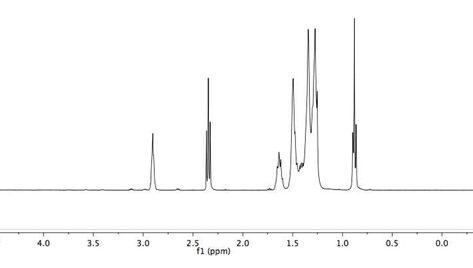
**

**Figure S4.** ^1^H NMR spectra for **4**

*Synthesis of epoxidized oleic acid,* ***4***^2^. To oleic acid (33.8 mL, 106.4 mmol) in toluene (180 mL) was added formic acid (12.3 mL, 326.0 mmol). The solution was stirred under reflux at 30 °C. H_2_O_2_ (72.1 mL, 2352.8 mmol) was added dropwise (over 1 hour) and the mixture left to stir for 24 hours. This was then transferred to a separation funnel and the organic phase was purified using NaHCO_3_ (sat. aq., 50 mL x 3), DI water (50 mL x 3) and dried with MgSO_4_ and filtered. The solution was then concentrated under reduced pressure to yield the product (**4**) as a white solid (26.5 g, 88.8 mmol, 84% yield).

**FTIR** (ATR) ν_max_ /cm^-1^: 2958, 2849, 1696, 1473, 1431, 1276, 1031, 1012, 846. **^1^H NMR** (400 MHz, CD_3_OD) δ_H_ 2.92 (m, 2H), 2.35 (t, *J* = 7.5, 2H), 1.65 (d, *J* = 6.9, 2H), 1.49 (dt, *J* = 6.9, 3.9, 4H), 1.35 (m, 10H), 1.29 – 1.27 (10H, m, 10H), 0.92 – 0.84 (m, 3H); **^13^C NMR** (101 MHz, CD_3_OD) δ_C_ 57.44, 57.39, 34.01, 32.00, 29,70, 29.68, 29.46, 29.32, 29.31, 29.09, 27.96, 27.92, 26.74, 26.70, 24.79, 22.81, 14.25; **HRMS** (ESI): Calculated for [C_13_H_20_NaO_3_]^+^ 320.47 obtained 321.24 (M^+^ Na^+^).

**10-(acryloyloxy)-9-hydroxyoctadecanoic acid (5)**

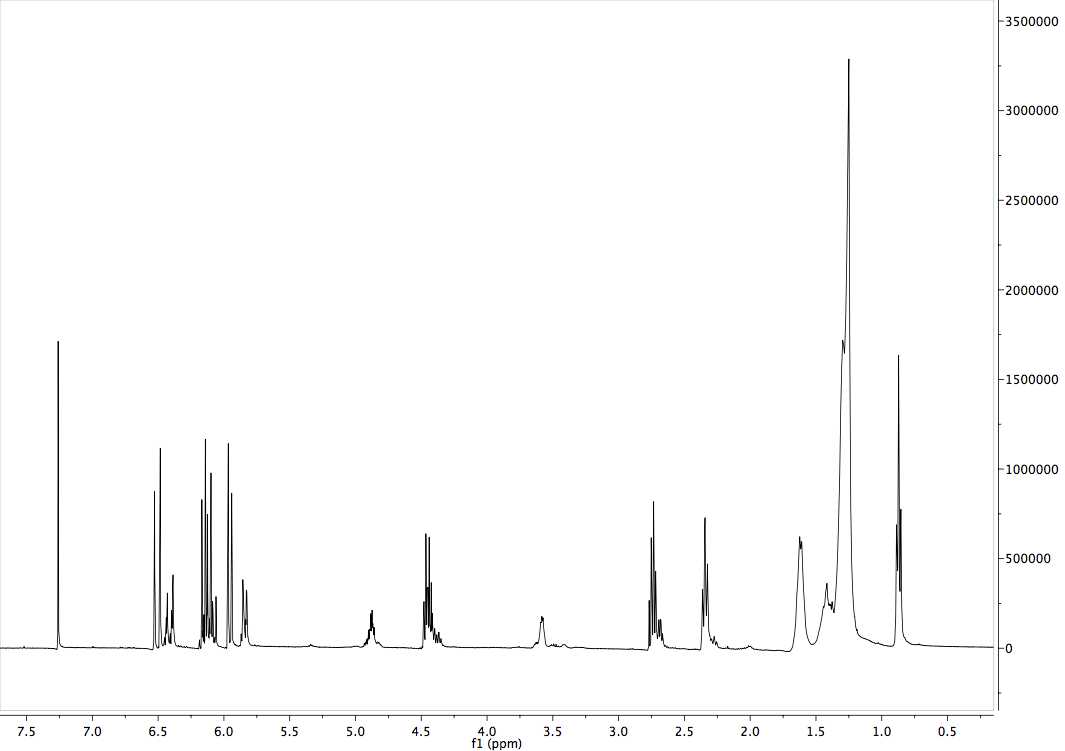


**Figure S5.** ^1^H NMR spectra for **5**

*Synthesis of acrylated oleic acid,* ***5***^2^. To a solution of **4** (40 g, 0.1 mmol) were added acrylic acid (acid with low H_2_O content, 99.5% stab. with ca. 200 ppm methoxyphenol, 76.12 mL, 1109.2 mmol) and hydroquinone (24 mg, 0.2 mmol). The reaction mixture was maintained at a mass ratio 2 : 1 acrylic acid : epoxidized oleic acid and left to stir for 6 hours at 100 °C. The aqueous layer was separated with diethyl ether (100 mL x 3) and the organic layer was washed with NaHCO_3_ (sat. aq., 50 mL x 3). The reaction mixture was then dried with MgSO_4_, filtered and concentrated to yield the title compound (**5**) as a whitish viscous liquid (48.2 g, 130.1 mmol, 97% yield).

**FTIR** (ATR) ν_max_ /cm^-1^: 3461, 2959, 2873, 1697, 1431, 1261, 1193, 771. **^1^H NMR** (400 MHz, CD_3_OD) δ_H_ 6.56 – 6.36 (m, 1H, H-20), 6.11 (ddd, *J* = 17.3, 15.2, 10.4, 1H, H-19), 5.96 (dd, *J* = 10.4, 1,4, 1H), 4.87 (m, 1H), 4.44 (tt, *J* 10.1, 6.3, 1H), 3.58 (m, 1H), 2.39 – 2.23 (m, 1H), 1.61 (m, 4H,), 1.43 (m, 2H), 1.30 (m, 10H), 1.26 (m, 10H), 0.91 – 0.80 (m, 3H); **^13^C NMR** (101 MHz, CD_3_OD) δ_C_ 171.10, 133.14, 131.53, 128.12, 128.07, 60.19, 34.08, 33.71, 31.99, 29.64, 29.39, 29.05, 25.50, 24.74, 22.80, 14.24; **HRMS** (ESI): Calculated for [C_13_H_20_NaO_3_]^+^ 392.57 obtained 393.26 (M^+^ Na^+^).

**Polymer TPA6**

**FTIR** (ATR) ν_max_ /cm^-1^: 3434 (-OH), 2931 (C-H), 1725 (C=O), 1448 (C-H), 1378 (-OH), 1245 (-OH), 1154 (C-O), 1025 (C-O), 943 (C=C), 914 (C=C), 840 (C=C), 814 (C=C); **^1^H NMR** (400 MHz, (CD_3_OD) δ_H_ 5.69 (br), 5.24 (br), 2.09 (br), 1.73 – 1.67 (br), 1.21 – 1.07 (br).

**Copolymer TPA7**

**FTIR** (ATR) ν_max_ /cm^-1^: 3434 (-OH), 2931 (C-H), 2856 (C-H), 1725 (C=O), 1448 (C-H), 1378 (-OH), 1245 (-OH), 1154 (C-O), 1025 (C-O), 943 (C=C), 914 (C=C), 840 (C=C), 814 (C=C); **^1^H NMR** (400 MHz, (CD_3_OD) δ_H_ 5.69 (br), 5.24 (br), 4.85 (br), 3.56 (br), 2.09 (br), 1.87 (br), 1.73 – 1.67 (br), 1.34 – 1.25 (br), 1.21 – 1.07 (br).

**a)**

**b)**

**Figure S6.** The polymer peaks from the GPC analyses of a) TPA6 and b) TPA7


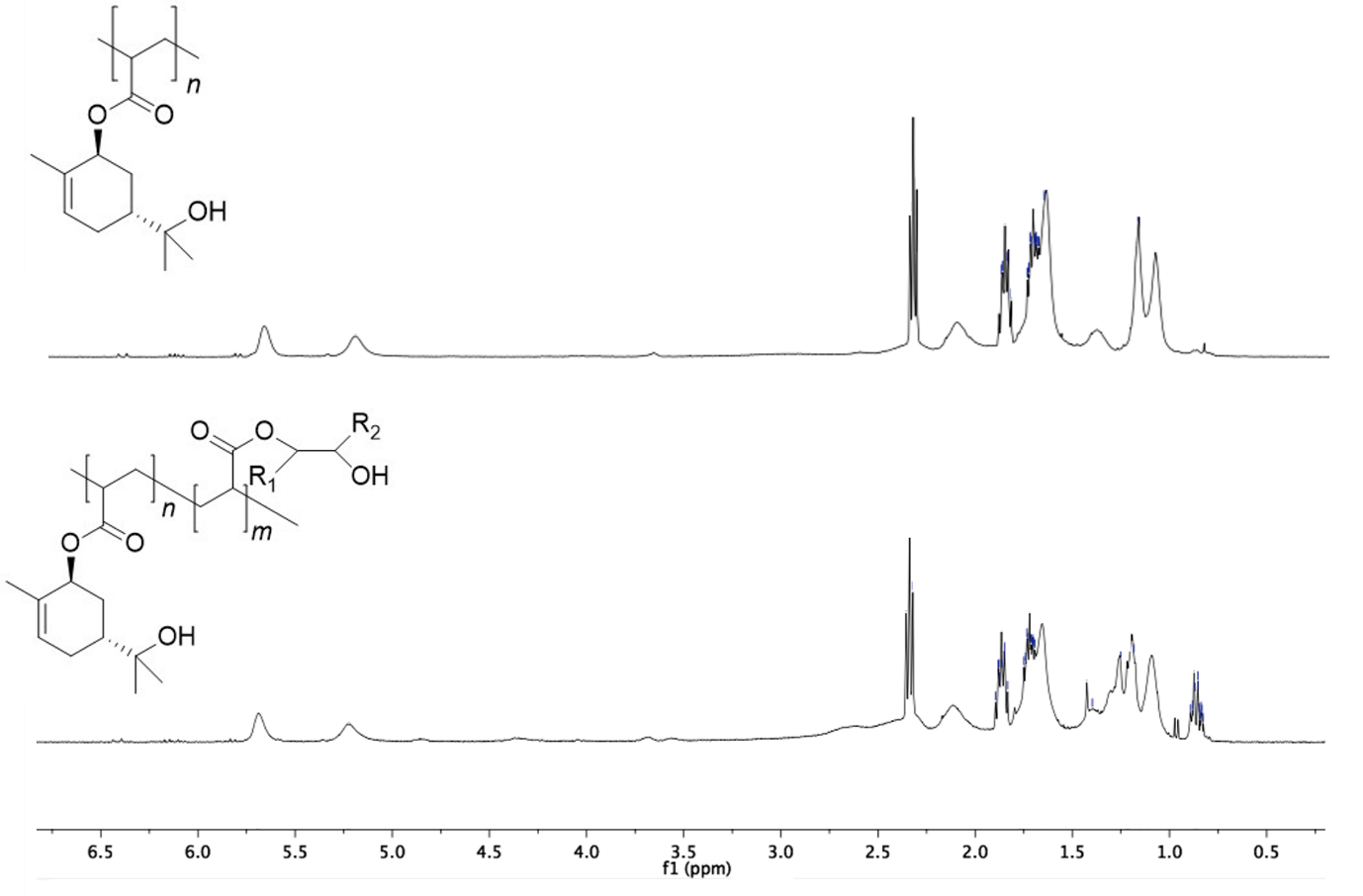


**Figure S7.** A comparison of the ^1^H NMR analyses of TPA6 and TPA7 after purification with hexane. The peaks at δ = 6.42 and 5.75 ppm representing the acrylate peaks no longer appeared, indicating that there were no residual monomer molecules left in the products.

**Table S1**. The *M*_w,app_ obtained from the sedimentation equilibrium experiment for all concentrations for TPA6.

| **Concentration (mg/mL)** | ***M*_w,app_ (*M**) (kDa)** | ***M*_w,app_ (hinge point) (kDa)** |
| --- | --- | --- |
| 0.5 | 3.3 | - |
| 0.75 | 3.8 | - |
| 1.0 | 4.1 | 3.6 |
| 1.5 | 3.0 | 3.2 |

**Table S2**. The *M*_w,app_ values obtained from the sedimentation equilibrium experiment for all concentrations for TPA7.

| **Concentration (mg/mL)** | | ***M*_w,app_ (*M**) (kDa)** | | ***M*_w,app_ (hinge point) (kDa)** | |
| --- | --- | --- | --- | --- | --- |
| 0.5 | 4.4 | 3.8 | |  |  |
| 0.75 | 4.0 | 3.9 | |  |  |
| 1.0 | 4.2 | 3.8 | |  |  |
| 1.5 | 4.7 | 4.3 | |  |  |
| 2.0 | 4.7 | 4.2 | |  |  |
| 3.0 | 4.6 | 4.1 | |  |  |
| 4.0 | 4.6 | 4.1 | |  |  |

**References**

1. Cutajar, M. *et al.* Terpene polyacrylate TPA5 shows favorable molecular hydrodynamic properties as a potential bioinspired archaeological wood consolidant. *Sci. Rep.* **11**, 7343 (2021).

2. Neto, W. S. *et al.* Superparamagnetic nanoparticles stabilized with free-radical polymerizable oleic acid-based coating. *J. Alloys Compd.* **739**, 1025–1036 (2017).
